# Supplementary material for: Resolving Symbiodiniaceae Diversity Across Coral Microhabitats and Reef Niches
Source: Environ Microbiol. 2025 Mar 4;27(3):e70065. doi: 10.1111/1462-2920.70065 (PMC11879917; doi:10.1111/1462-2920.70065)
Supplement: Supplementary file 1 — Data S1. Supporting Information. [file EMI-27-e70065-s001.docx]

**APPENDIX**

**Table S1:**  Summary of the average number of reads per sample for each sample type calculated with sample sizes after removing three samples with less than 15,000 reads as stated in the methods.

| **Sample Source** | **Average number of reads per sample** |
| --- | --- |
| Coral Tissue | 403,403 |
| Coral Mucus | 173,621 |
| Seawater | 251,571 |
| Turf Algae | 119,590 |
| Near-reef Sediment | 151,253 |
| Distant Sediment | 98,218 |

**Table S2**: Summary of results of the PERMANOVA (adonis2) comparing overall community composition among environment types (seawater, near-reef and distant sediment, turf algae, SML, and tissue) based on Bray Curtis Dissimilarity.

| **Factor** | **DF** | **Sum of Squares** | **R2** | **F** | **Pr (>F)** |
| --- | --- | --- | --- | --- | --- |
| environment type | 5 | 10.947 | 0.15392 | 6.760 | 0.001 |
| plot | 4 | 1.342 | 0.01887 | 1.036 | 0.376 |
| environment type × plot | 20 | 5.069 | 0.07127 | 0.783 | 0.982 |
| Residual | 166 | 53.764 | 0.75594 |  |  |
| Total | 195 | 71.122 | 1 |  |  |

**Table S3**: Pairwise adonis comparing overall ITS2 community based on Bray Curtis Dissimilarity of each habitat. Adjusted p-value represents Bonferroni & Hochberg multiple test corrected p-value.

| **Pairs** | **F.Model** | **R2** | **P-value** | **Adjusted P-value** |
| --- | --- | --- | --- | --- |
| SML vs tissue | 0.06610665 | 0.00068105 | 1 | 1 |
| SML vs turf algae | 4.43616687 | 0.05016236 | 0.002 | 0.002307692 |
| SML vs distant sediment | 8.32493016 | 0.11052025 | 0.001 | 0.001666667 |
| SML vs near-reef sediment | 15.6134325 | 0.18899387 | 0.001 | 0.001666667 |
| SML vs seawater | 5.1741059 | 0.07168923 | 0.002 | 0.002307692 |
| tissue vs turf algae | 4.5182762 | 0.05047323 | 0.002 | 0.002307692 |
| tissue vs distant sediment | 8.33755081 | 0.10921952 | 0.001 | 0.001666667 |
| tissue vs near-reef sediment | 15.6362111 | 0.18695504 | 0.001 | 0.001666667 |
| tissue vs seawater | 5.29110851 | 0.07219305 | 0.002 | 0.002307692 |
| turf algae vs distant sediment | 4.78247398 | 0.07999793 | 0.001 | 0.001666667 |
| turf algae vs near-reef sediment | 8.11487506 | 0.1285731 | 0.001 | 0.001666667 |
| turf algae vs seawater | 4.69162905 | 0.07859777 | 0.001 | 0.001666667 |
| distant sediment vs near-reef sediment | 2.72306359 | 0.06686785 | 0.003 | 0.003214286 |
| distant sediment vs seawater | 9.17824191 | 0.19454396 | 0.001 | 0.001666667 |
| near-reef sediment vs seawater | 18.258485 | 0.32454633 | 0.001 | 0.001666667 |

**Table S4**: Summary of ANOVA (*vegan*) comparing the homogeneity of habitat type variances (i.e. dissimilarity of samples within a given compartment) based on Bray Curtis Dissimilarity

|  | **Df** | **Sum of Squares** | **Mean Squares** | **F value** | **Pr (>F)** |
| --- | --- | --- | --- | --- | --- |
| Groups | 5 | 0.795 | 0.159006 | 2.9377 | 0.01404 |
| Residuals | 190 | 10.284 | 0.054125 |  |  |

**Tables S5**: Summary of permutation test for homogeneity of multivariate dispersions for all pairwise comparisons of habitat types. Observed p-values are below the diagonal, permuted p-values are above the diagonal. Average distance to the median, as calculated by betadisper in *vegan*, for each habitat is included in parentheses.

|  | **Seawater** | **Neer-reef Sediment** | **Distant Sediment** | **SML** | **Tissue** | **Turf algae** |
| --- | --- | --- | --- | --- | --- | --- |
| **Seawater (0.366)** |  | 0.025 | 0.001 | 0.022 | 0.028 | 0.001 |
| **Near-reef Sediment (0.463)** | 0.023 |  | 0.101 | 0.356 | 0.356 | 0.001 |
| **Distant Sediment (0.541)** | 0.000 | 0.114 |  | 0.846 | 0.853 | 0.080 |
| **Coral Mucus (0.5266)** | 0.023 | 0.372 | 0.844 |  | 0.982 | 0.171 |
| **Tissue (0.528)** | 0.024 | 0.367 | 0.862 | 0.980 |  | 0.184 |
| **Turf algae (0.601)** | 0.000 | 0.000 | 0.080 | 0.150 | 0.164 |  |

**Table S6**: Number of unique DIVs (recorded in Symportal database) present in each habitat type and the average found in any given sample as well as the distribution of a habitat’s DIVs across Symbiodiniaceae genera.

|  | **No. of unique DIVs** | **Average per sample** | ***Sym.*** | ***Clad.*** | ***Dur.*** | ***Fug.*** | ***Ger.*** | ***Hall.*** |
| --- | --- | --- | --- | --- | --- | --- | --- | --- |
| seawater | 229 | 42.95 | 90 | 97 | 31 | 0 | 10 | 1 |
| turf algae | 207 | 16 | 115 | 74 | 18 | 0 | 0 | 0 |
| near-reef sediment | 140 | 21.2 | 71 | 49 | 17 | 1 | 1 | 1 |
| distant sediment | 169 | 21.05 | 57 | 78 | 24 | 1 | 8 | 1 |

**Table S7**: List of ITS2 sequences shared between all free-living and coral habitats

| A1 | *Symbiodinium* | D3a | *Durusdinium* | X56421_A | *Symbiodinium* |
| --- | --- | --- | --- | --- | --- |
| C1 | *Cladocopium* | D4 | *Durusdinium* | X56861_D | *Durusdinium* |
| C15 | *Cladocopium* | D4c | *Durusdinium* | X8111_A | *Symbiodinium* |
| C1b | *Cladocopium* | X4185_A | *Symbiodinium* | X8182_A | *Symbiodinium* |
| C41 | *Cladocopium* | X4935_D | *Durusdinium* | X9354_A | *Symbiodinium* |
| D1 | *Durusdinium* | X56419_A | *Symbiodinium* |  |  |

**Table S8**: List of non-matching tissue and mucus samples and their corresponding dominant ITS2 type profile

| **Source** | **Coral #** | **Coral Genus** | **Sample.ID** | **Dominant Type Profile** |
| --- | --- | --- | --- | --- |
| coral | 21 | Acropora | Coral_ITS_021 | C41-C1-C41b-C41j-C41g-C41w |
| mucus | 21 | Acropora | Mucus_ITS_041 | C41/C1-C41b-C41j-C1b |
| coral | 31 | Acropora | Coral_ITS_031 | C41/C1-C41b-C1b-C41o |
| mucus | 31 | Acropora | Mucus_ITS_050 | C41-C1-C41x-C41b-C1b |
| coral | 38 | Acropora | Coral_ITS_038 | C41/C1-C41b-C41j-C1b |
| mucus | 38 | Acropora | Mucus_ITS_057 | C41-C1-C41b-C41j-C41g-C41w |
| coral | 40 | Acropora | Coral_ITS_040 | C41/C1-C41b-C41j-C1b |
| mucus | 40 | Acropora | Mucus_ITS_059 | C41-C1-C41x-C41b-C1b |
| coral | 46 | Acropora | Coral_ITS_046 | C41-C1-C41x-C41b-C1b |
| mucus | 46 | Acropora | Mucus_ITS_065 | C41-C1-C41b-C41j-C41g-C41w |
| coral | 36 | Goniastrea | Coral_ITS_036 | C41-C1-C41b-C41j-C41g-C41w |
| mucus | 36 | Goniastrea | Mucus_ITS_055 | C41-C1-C41x-C41b-C1b |
| coral | 4 | Platygyra | Coral_ITS_004 | C41/C1-C41b-C41j-C1b |
| mucus | 4 | Platygyra | Mucus_ITS_024 | C41-C1-C41b-C41j-C41g-C41w |
| coral | 8 | Echinopora | Coral_ITS_008 | D1-D4-D17d-D4c-D17e-D6-D1r-D17c-D17j |
| mucus | 8 | Echinopora | Mucus_ITS_028 | D1-D4c-D4-D6-D1h |
| coral | 50 | Fungia | Coral_ITS_050 | C1/C39-C1b-C41-C1ae-C41f-C41a-C1eb |
| mucus | 50 | Fungia | Mucus_ITS_069 | C1/C41e-C1b-C41f-C41a-C41-C39-C1ae-C1f-C1eb |
| coral | 25 | Ctenactis | Coral_ITS_025 | C1/C41e-C1b-C41f-C41a-C41-C39-C1ae-C1f-C1eb |
| mucus | 25 | Ctenactis | Mucus_ITS_045 | C41/C1-C41b-C41j-C1b |
| coral | 23 | Pavona | Coral_ITS_023 | D1-D4-D4c-D1h-D1c-D2 |
| mucus | 23 | Pavona | Mucus_ITS_043 | C1-C1b-C39-C41-C41f-C41a-C1ae-C41e-C1af-C1f-C39a |
| coral | 12 | Pocillopora | Coral_ITS_012 | A1/A1c-A1h-A1cc-A1ce-A1ch |
| mucus | 12 | Pocillopora | Mucus_ITS_032 | A1-A1cc-A1c-A1h-A1q-A1i |
| coral | 9 | Pocillopora | Coral_ITS_009 | A1-A1c-A1cc-A1h-A1bv-A1cb-A1ce |
| mucus | 9 | Pocillopora | Mucus_ITS_029 | A1-A1cc-A1c-A1h-A1q-A1i |
| coral | 17 | Millepora | Coral_ITS_017 | A1/A1k-A1g-A1b |
| mucus | 17 | Millepora | Mucus_ITS_037 | A1k/A1-A1ea |
| coral | 22 | Millepora | Coral_ITS_022 | A1k/A1-A1ea |
| mucus | 22 | Millepora | Mucus_ITS_042 | A1/A1k-A1b-A1z |
| coral | 20 | Unknown | Coral_ITS_020 | D1-D4-D4c-D1h-D1c-D2 |
| mucus | 20 | Unknown | Mucus_ITS_040 | D1-D4-D4c-D4f-D1b-D1h |


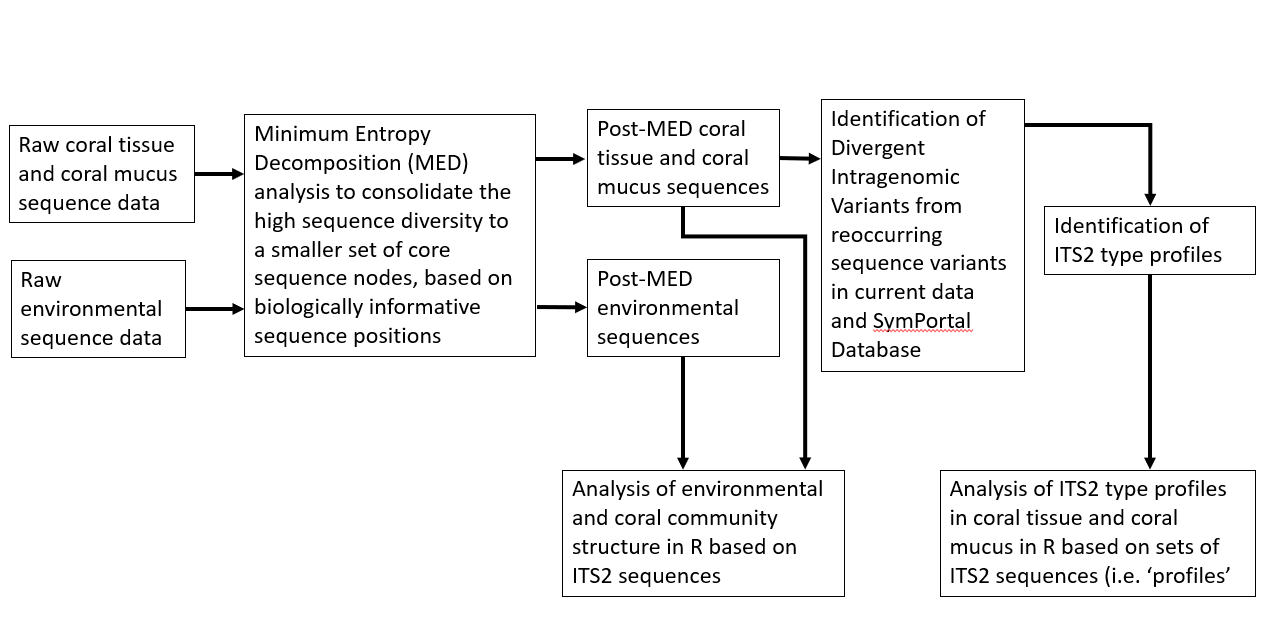


**Figure S1**: Workflow to visualize how coral-based and environment-based samples were analyzed here. Specifically, the coral and environmental samples were run as 2 distinct SymPortal runs, with the difference that environmental samples were not subjected to ITS2 type profile discovery because they violate the basic “one-dominant-profile” assumption used by SymPortal to identify intragenomic vs intergenomic variation.


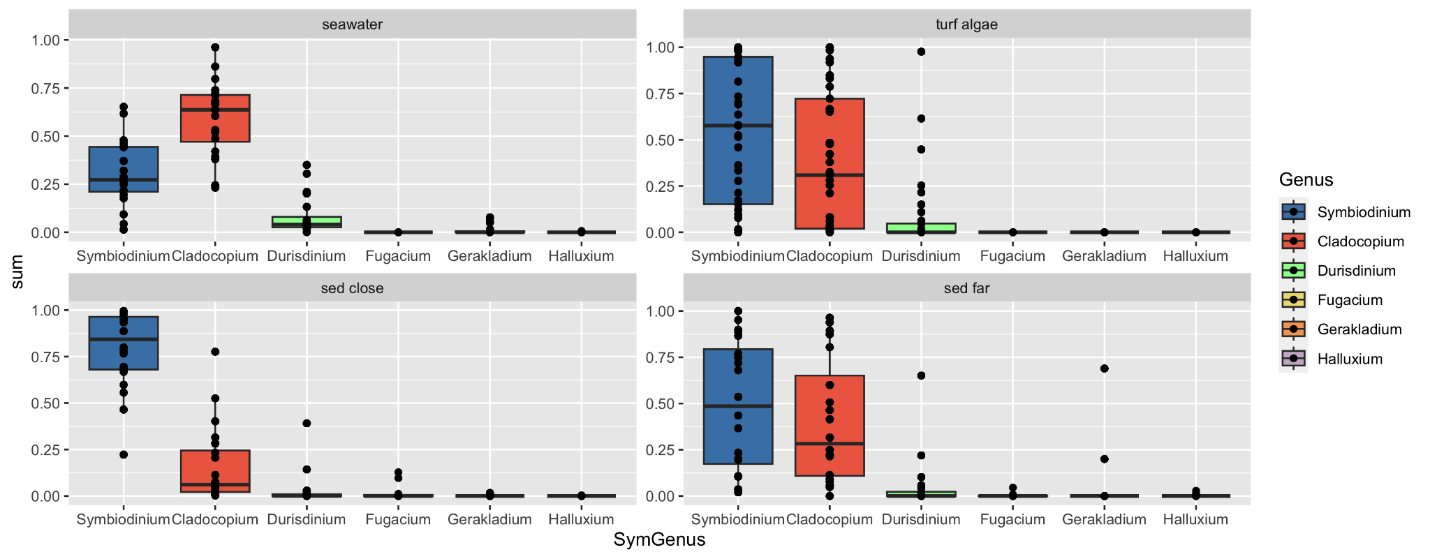


**Figure S2**: Distribution of genus-level relative abundance within samples from each free-living habitat.


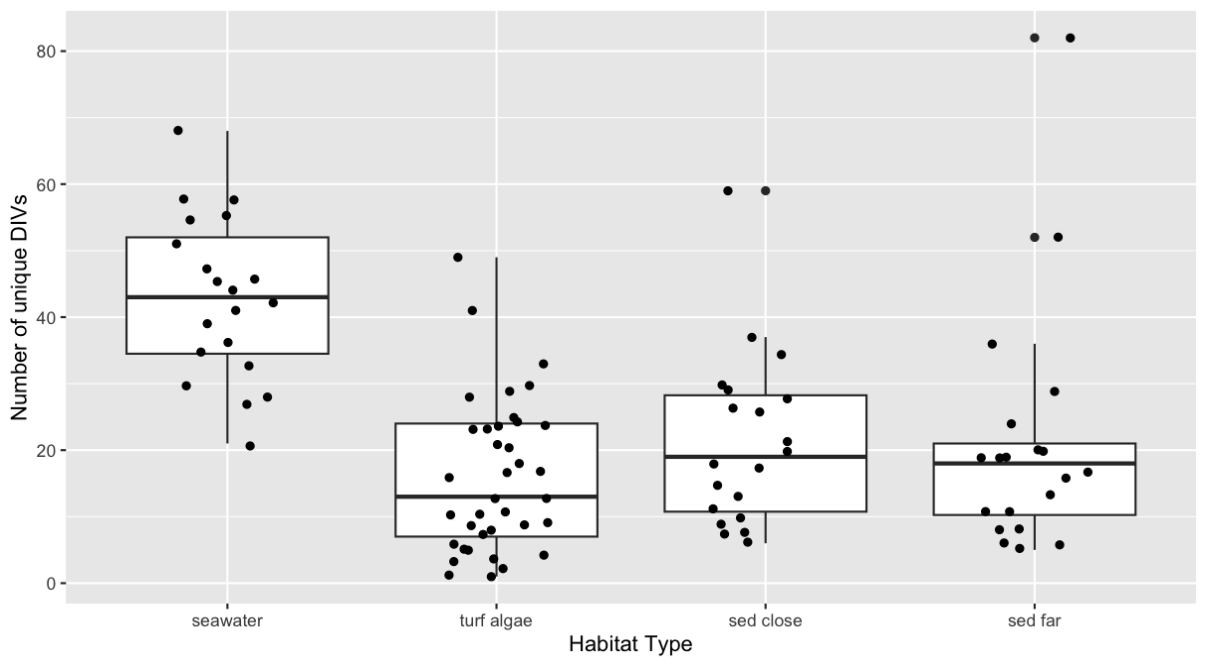


**Figure S3**: Distribution of unique DIVs per sample within each free-living habitat type


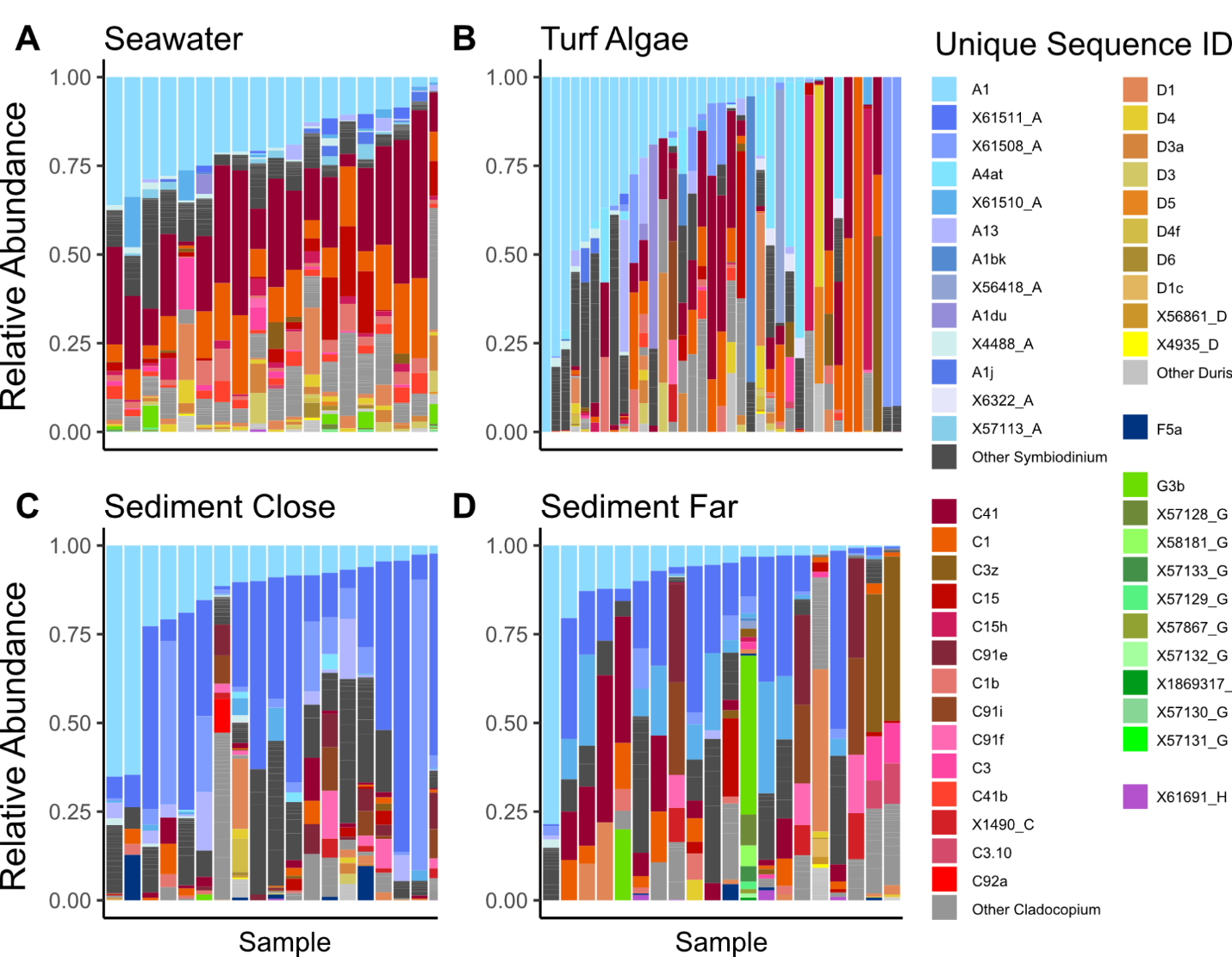


**Figure S4**: Relative abundance of unique DIVs in samples from (A) seawater (A), turf algae (B), near-reef sediment (C), and distant sediment (D) environmental sources. Sequences representing less than 1% of the total abundance of sequences per Symbiodiniaceae genus across all samples are grouped as “Other”. Two sequences, A13 and A1du, were highly abundant in single samples despite being grouped as “Other *Symbiodinium*” so these sequences were given a distinct label.


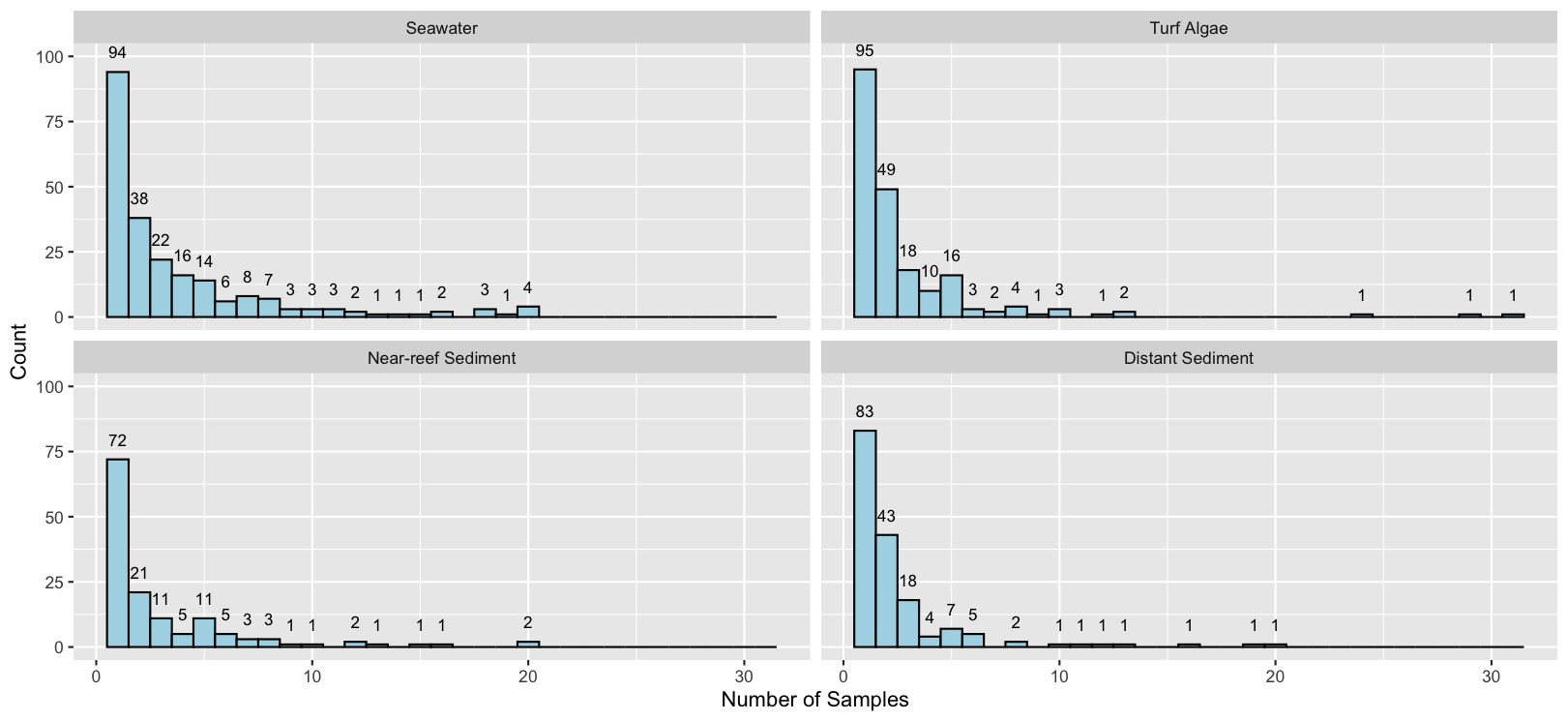


**Figure S5**: Histogram showing the frequency distribution of distinct DIVs for a given number of samples.


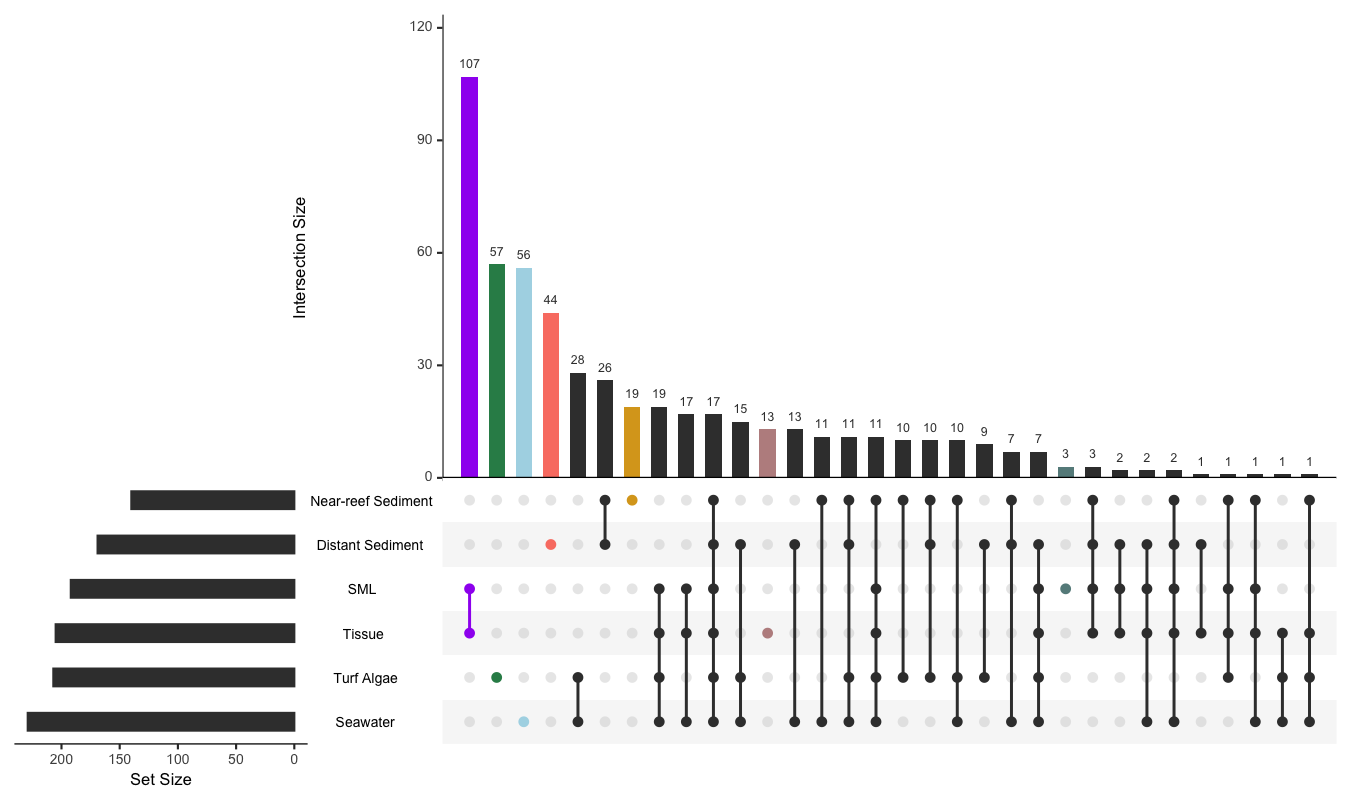


**Figure S6**: Distribution of shared ITS2 sequences between combinations of coral and non-coral sources. The number of sequences shared between a given set of sources (distinguished by filled points) is displayed by the vertical barplot. Total number of unique sequences for each source is shown in the horizontal bar plot.


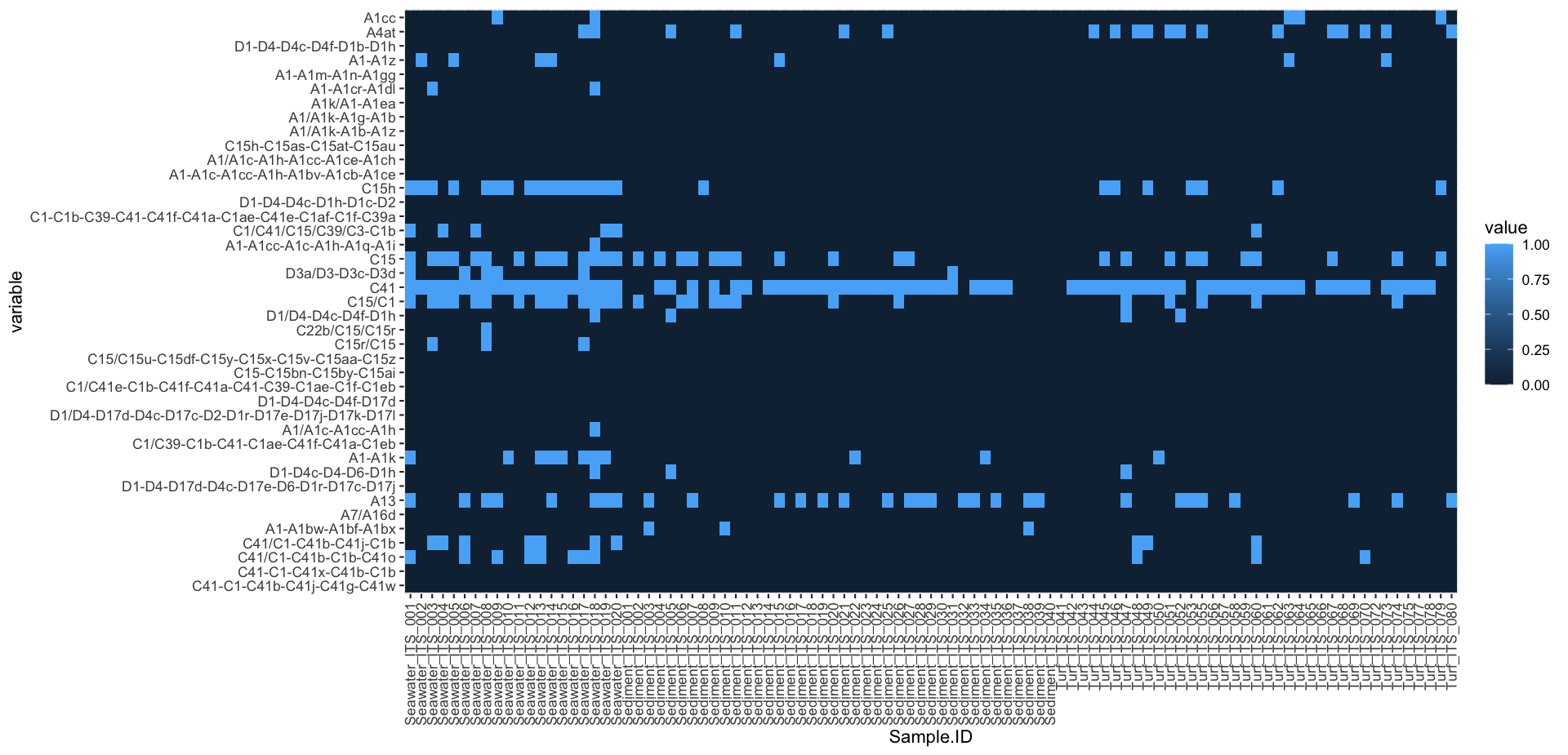


**Figure S7**: Presence/absence of complete DIV sets within environmental samples (columns) corresponding to the ITS2 type profiles (rows) found in coral samples. Light blue indicated all DIVs in the given ITS2 type profile were reported in the given sample. Dark blue indicates that at least one DIV was not present in that sample.


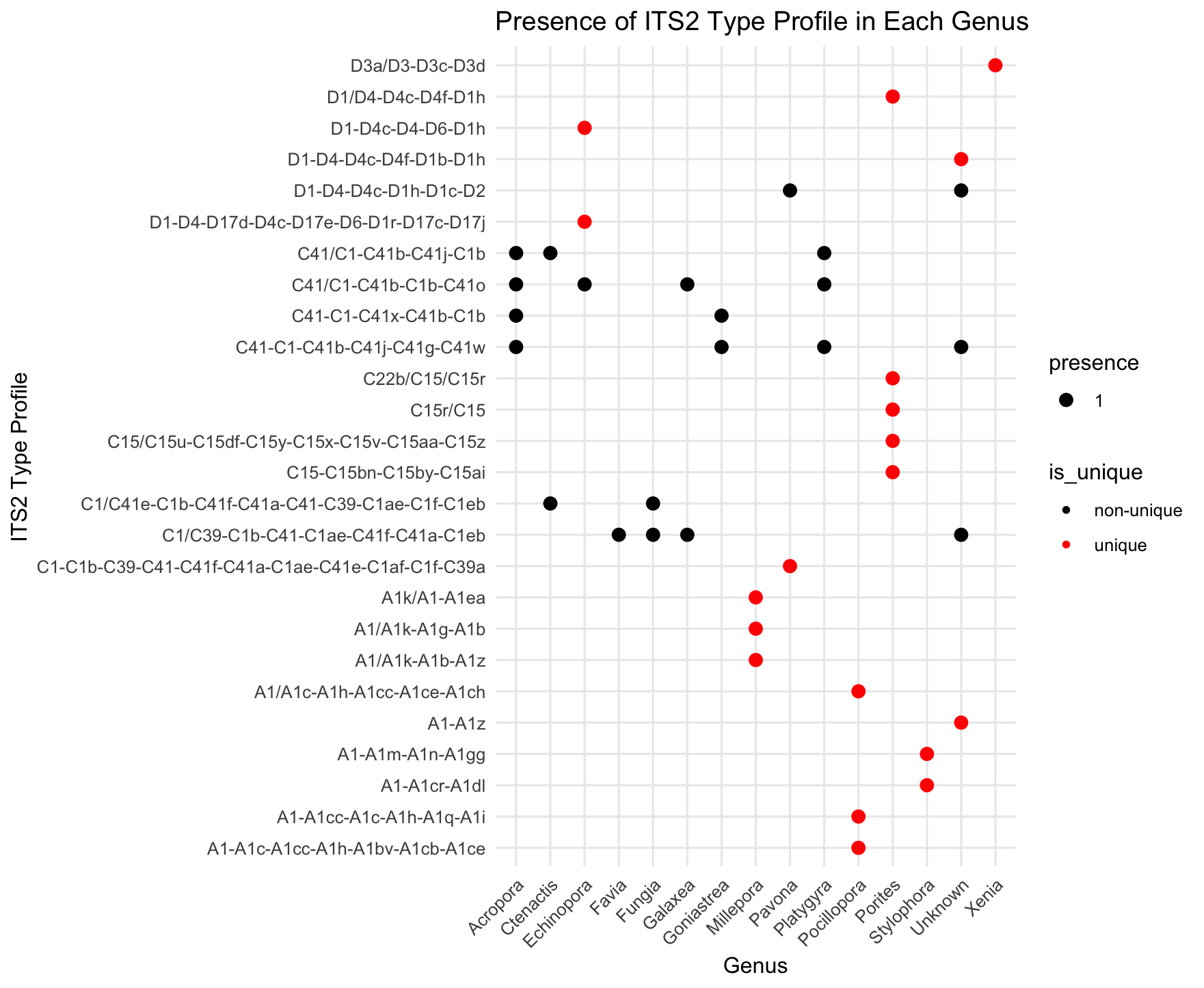


**Figure S8:** Presence of dominant ITS2 type profiles (>50% relative abundance within a sample, n=26) across coral genera assessed here. Both tissue and mucus samples are included for each genus - allowing genera with a single coral colony sampled (e.g. *Pavona*) to return two different dominant ITS2 type profiles. Red points show ITS2 type profiles that are unique to a single genus while black points show ITS2 type profiles present across genera.


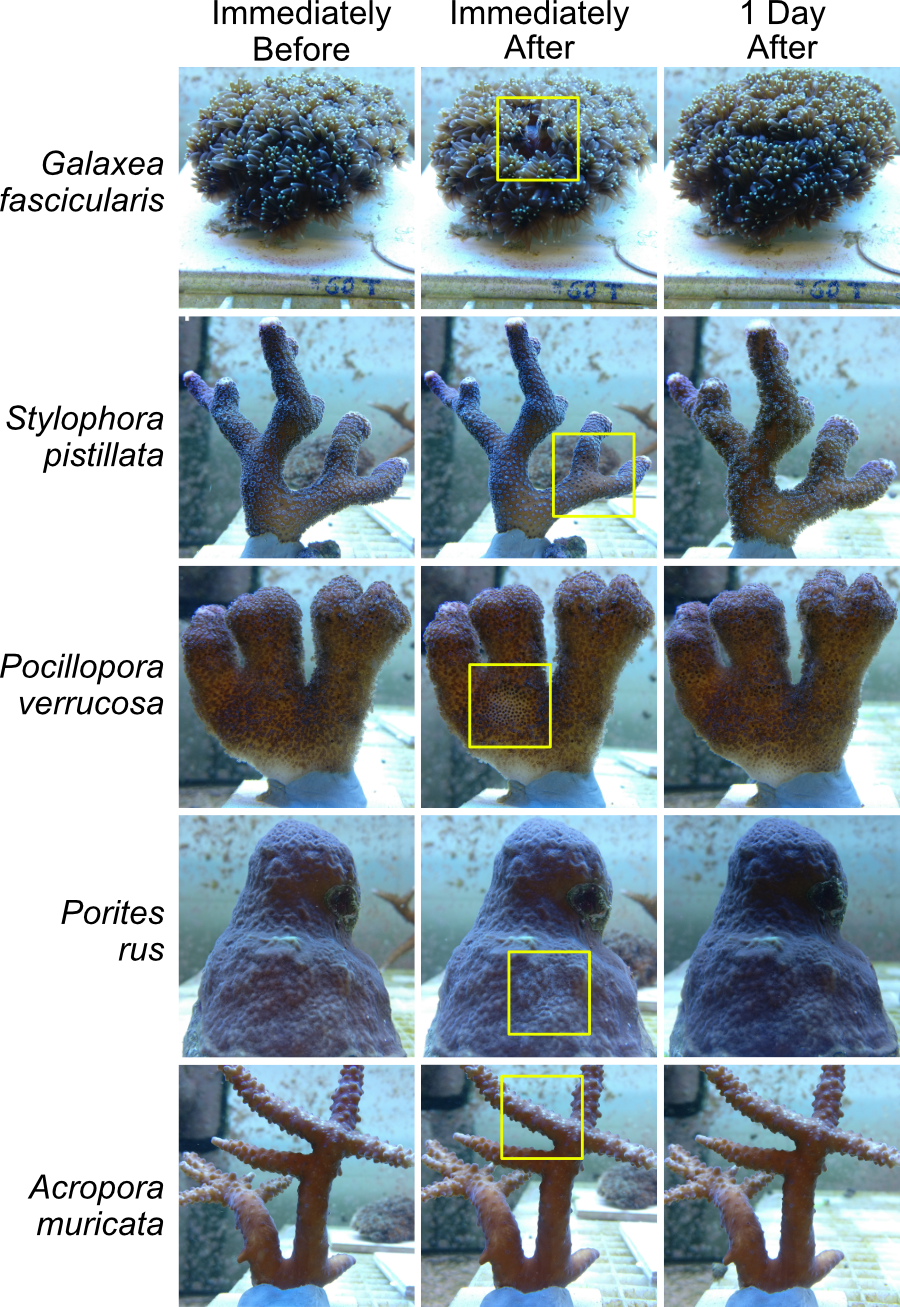


**Figure S9**: Resepentative colonies of each of 5 coral species subjected to simulated mucus sampling and their condition prior to, immediately after, and one day post sampling. The sampled area on each colony is demarcated with a yellow square.
